# New Horizons in cellular senescence for clinicians

**DOI:** 10.1093/ageing/afad127

**Published:** 2023-07-15

**Authors:** Miles D Witham, Antoneta Granic, Satomi Miwa, Joao F Passos, Gavin D Richardson, Avan A Sayer

**Affiliations:** AGE Research Group, Translational and Clinical Research Institute, Faculty of Medical Sciences, Newcastle University, Newcastle, UK; NIHR Newcastle Biomedical Research Centre, Newcastle upon Tyne Hospitals NHS Foundation Trust, Cumbria, Northumberland, Tyne and Wear NHS Foundation Trust and Newcastle University, Newcastle, UK; AGE Research Group, Translational and Clinical Research Institute, Faculty of Medical Sciences, Newcastle University, Newcastle, UK; NIHR Newcastle Biomedical Research Centre, Newcastle upon Tyne Hospitals NHS Foundation Trust, Cumbria, Northumberland, Tyne and Wear NHS Foundation Trust and Newcastle University, Newcastle, UK; Biosciences Institute, Faculty of Medical Sciences, Newcastle University, Newcastle, UK; Department of Physiology and Biomedical Engineering and Robert and Arlene Kogod Center on Aging, Mayo Clinic, Rochester, MN, USA; Vascular Medicine and Biology Theme, Biosciences Institute, Faculty of Medical Sciences, Newcastle University, Newcastle, UK; AGE Research Group, Translational and Clinical Research Institute, Faculty of Medical Sciences, Newcastle University, Newcastle, UK; NIHR Newcastle Biomedical Research Centre, Newcastle upon Tyne Hospitals NHS Foundation Trust, Cumbria, Northumberland, Tyne and Wear NHS Foundation Trust and Newcastle University, Newcastle, UK

**Keywords:** cellular senescence, senescence-associated secretory phenotype, senolytics, senotherapeutics, human ageing, interventions, older people

## Abstract

Cellular senescence has emerged as a fundamental biological mechanism underpinning the ageing process and has been implicated in the pathogenesis of an increasing number of age-related conditions. Cellular senescence is a cell fate originally defined as an irreversible loss of replicative potential although it is now clear that it can be induced by a variety of mechanisms independent of replication and telomere attrition. The drivers include a persistent DNA damage response causing multiple alterations in cellular function. Senescent cells secrete a range of mediators that drive chronic inflammation and can convert other cells to the senescent state—the senescence-associated secretory phenotype. Much research to date has been conducted in animal models, but it is now clear that senescent cells accompany ageing in humans and their presence is an important driver of disease across systems. Proof-of-concept work suggests that preventing or reversing senescence may be a viable strategy to counteract human ageing and age-related disease. Possible interventions include exercise, nutrition and senolytics/senostatic drugs although there are a number of potential limitations to the use of senotherapeutics. These interventions are generally tested for single-organ conditions, but the real power of this approach is the potential to tackle multiple age-related conditions. The litmus test for this exciting new class of therapies, however, will be whether they can improve healthy life expectancy rather than merely extending lifespan. The outcomes measured in clinical studies need to reflect these aims if senotherapeutics are to gain the trust of clinicians, patients and the public.

## Key Points

- The accumulation of senescent cells is a fundamental biological mechanism underlying ageing and age-related conditions.
- Senescent cells secrete a range of mediators that drive chronic inflammation and can convert other cells to the senescent state.
- Interventions that remove senescent cells (senolytics) or that block the deleterious effects of senescent cells (senostatics) hold promise as ways to treat multiple age-related conditions.
- Clinical trials are needed to test whether the promise of senotherapeutic interventions translates into benefit for older people.

## Introduction

Cellular senescence has emerged as one of the fundamental biological mechanisms underpinning the ageing process. It has been implicated in the pathogenesis and progression of an increasing number of age-related conditions, and excitingly, interventions that are able to halt the progression of senescence, remove senescent cells or mitigate the effects of cellular senescence are now reaching the stage of testing in clinical trials. Such therapies hold out the promise of tackling multiple age-related conditions simultaneously. In this New Horizons review, we provide an accessible overview of the biology of senescence, its relevance to age-related conditions and the current state of play in terms of interventions to tackle cellular senescence and its consequences.

## What is cellular senescence?

Cellular senescence is a cell fate originally defined as an irreversible loss of replicative potential [1]. Cells are able to replicate only a finite number of times [2]; this limit (the ‘Hayflick limit’) was first described in the 1960s. More recently, the key role of telomere shortening in this process has been recognised—each cell division is accompanied by telomere shortening until the non-replication threshold is reached.

However, it is now evident that cellular senescence can also be induced by a variety of mechanisms independently of replication and telomere attrition. Cellular damage (e.g. DNA, protein and lipid damage from toxins or radiation) can trigger senescence, and importantly, inflammatory mediators (driven by infections such as COVID-19 [3], tissue damage such as chronic wounds, or autoimmune conditions) can also trigger senescence. The discovery of a wide range of different markers of senescence above and beyond cell cycle arrest has also widened our view of what constitutes a senescent cell. It is now clear that post-mitotic (non-dividing) cells including myocytes, neurons, osteocytes and adipocytes [4–7] can also become senescent. A hallmark of senescent cells is their manifestation of a pro-inflammatory phenotype, termed the ‘senescence-associated secretory phenotype’ (SASP) discussed in more detail below. This phenotype contributes to tissue dysfunction, driving chronic inflammation and promoting tissue remodelling [8]. Importantly, because inflammatory mediators produced by senescent cells can induce senescence, senescence can be locally ‘contagious’—a senescent cell can induce senescence in neighbouring cells via the SASP [9]; a phenomenon sometimes referred to as the bystander effect.

## The cellular consequences of senescence

**Figure 1:**
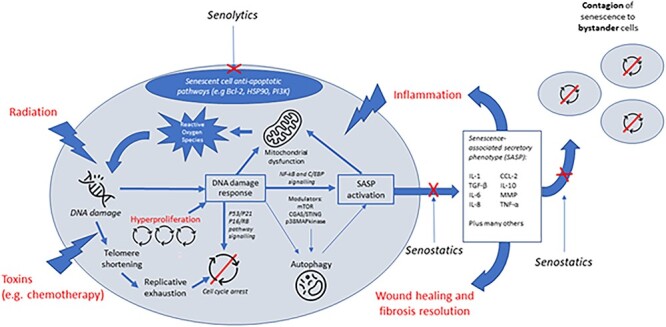
Cellular senescence: mechanisms and potential points of intervention.

A persistent DNA damage response (DDR) is a consistently observed feature of senescent cells and causes multiple alterations in cellular function. The DDR is triggered by short telomeres or by damage to DNA. The DDR pathways first initiate cell cycle arrest, and when they are persistent, they also induce the SASP [10, 11]. Other signalling pathways that regulate the SASP are also altered in senescent cells; these include the p38 MAP kinase, CGAS/STING and, importantly, the mechanistic/mammalian Target of Rapamycin (mTOR) pathways. The SASP comprises a variety of secreted factors including pro-inflammatory cytokines such as interleukin-6, chemokines, growth factors and matrix-modifying factors (Figure 1) that are typically driven by increased activity of nuclear factor kappa B (NF-κB) and CCAAT enhancer sequence binding proteins. Both the DDR and the SASP can also initiate senescence-associated mitochondrial dysfunction, impairing energy generation and increasing the generation of reactive oxygen species [12, 13], which then further damage cellular structures (e.g. lipid envelopes) and macromolecules including DNA. Damaged mitochondria are usually removed by the process of mitophagy (mitochondria-specific autophagy), but this process too is downregulated in senescent cells [14], leading to a vicious cycle of further reactive oxygen species generation, DNA damage and DDR. A range of other alterations have also been observed in senescent cells, including persistent mTOR activation [15–17], alterations to autophagy [18–22], epigenetic reprogramming and reorganisation of chromatin [23, 24]. These cellular changes lead to organ-level dysfunction via inflammation and immune cell infiltration, loss of functional cells contributing to organ activity and structural disruption, for example, because of fibrosis and fatty infiltration.

## Evidence for senescence in human ageing

Much of the research conducted on cellular senescence to date has been conducted in non-human cell, organ or whole-body models. It is now clear, however, that senescent cells accompany ageing in humans and their presence is an important driver of disease. Furthermore, senescence is a prominent feature of genetically driven premature human ageing syndromes such as dyskeratosis congenita and Hutchison–Gilford progeria syndrome [25, 26]. Analysis of senescent cell accumulation during typical human ageing is challenging, as the senescent phenotype is heterogenous [27], and to date, no unique or universal markers of all senescent cells have been identified [28]. As such, senescent cells *in vivo* are often recognised via analysis of several senescence-associated characteristics including the expression of the cell-cycle regulators (p16INK4a, p21Cif/waf, p53), the presence of DNA damage markers, increased lysosomal activity (senescence-associated β-galactosidase staining, SA-β-Gal) and the absence of proliferation markers. Despite the challenges, it is now generally accepted that senescent cells accumulate in a wide range of human tissues with advancing age and also accumulate in tissues with age-related pathophysiology. A recent systematic review of 103 articles [29] confirmed that although not all senescent cell markers have been investigated in all tissues, there is sufficient evidence to demonstrate that increased senescence is observed in a wide range of age-related diseases, including diseases affecting the brain, cardiovascular system, respiratory system, liver, kidneys and immune system. Whilst the expression of the SASP in human tissues across the lifespan is less well studied, several candidate SASP proteins correlate with chronological age and with biomarkers of ageing in human plasma [30, 31].

## Senescence as a driver of age-related conditions

### Immunological

Immunosenescence [32] leads to the impaired ability of older adults to respond to infections and vaccinations [33] and is associated with an increased risk of age-related chronic diseases [29, 34]. A complex set of changes in both T- and B-cell repertoire has been described [35]. There is a marked decrease in naïve T and B cells in the circulation with advancing age, with decreased cellular diversity and clonal distribution [36]. The reduction in B-cell repertoire impairs production of high-affinity protective antibodies [33]. In parallel, life-long exposure to pathogens (including viruses such as cytomegalovirus) leads to increased pools of memory T cells with a late-differentiated and pro-inflammatory phenotype. This population includes several subsets of T cells with senescent-like phenotypic features including low CD-28 surface expression, replicative senescence, shorter telomeres and increased production of pro-inflammatory cytokines, for example, IL-4, IL-5 and IFNγ [33, 35, 37].

Immunosenescence and immune ageing are particularly important aspects of the cellular senescence paradigm because of the key role of immune dysfunction in multiple other age-related conditions. This is particularly true for those conditions with inflammatory or accelerated ageing phenotypes [34, 37]. Senescent immune cells have been implicated in the pathogenesis or consequences of atherosclerotic coronary artery disease, rheumatoid arthritis and type 2 diabetes mellitus; other examples are given in the sections below.

### Neurological

Cellular senescence and SASP-mediated inflammation have been implicated as biological mechanisms of brain ageing and neurodegeneration observed in both Alzheimer’s disease and tauopathies [38–40]. Co-localisation of senescence markers and expression of multiple SASP genes reported in mouse models of Alzheimer’s dementia (AD) have been confirmed in human neurons bearing neurofibrillary tangles. These neurons showed an elevation of markers of cell cycle arrest (p16 and p53) and DDR (γH2A.X), and an increase in SASP transcripts (NF-κB, IL-β, CXCL1) [41].

Mechanistic studies *in vitro* with human astrocytes have revealed premature stress-induced senescence and elevation of classic senescent markers (SA-β-Gal activity and upregulation of p53, p21 and p16); these changes are hypothesised to further exacerbate AD pathology (amyloid-β (Aβ) plaques and neurofibrillary tangle burden), neuronal dysfunction and compromised blood–brain barrier [42]. Conversely, AD pathology triggers senescence, reinforcing the accumulation of senescent cells and SASP by creating a positive feedback loop and driving further cellular senescence in multiple brain cell types [39]. Recent studies confirm that senolytic drugs can reduce senescent cells and brain inflammation, and improve cognitive function, in mouse models of brain ageing [43].

### Musculoskeletal

Senescence markers and the SASP are present in aged mouse skeletal muscle cells, but such findings have not been easy to replicate in human muscle tissue analyses; studies across the range of human ageing are needed. Whilst some early analyses of human muscle from older people found an increase in mRNA levels of the senescence-associated genes involved in cell cycle arrest/DNA damage [44], others failed to observe an elevation of senescence markers in muscle nuclei [45, 46]. A recent comprehensive assessment of human skeletal muscle biopsies from middle-aged and older people found evidence that senescence does appear to be a feature of human muscle ageing [46]. PCR of single myofibers showed increased p16 and p21 mRNA expression in older muscle, along with increased staining for the DNA-damage response protein γH2A.X.

Subsets of cells in the bone microenvironment also exhibit a senescence phenotype and secrete the SASP in animal models [47] and in human bone biopsies. The extent to which senescence and the accompanying SASP are drivers of osteoporosis is unclear, however. Similar findings have been highlighted in models of osteoarthritis—senescent chondrocytes are found in osteoarthritis cartilage, the introduction of senescent chondrocytes can induce osteoarthritic changes and senolytic drugs can ameliorate the development of osteoarthritis after trauma in animal models [48].

### Cardiovascular

Senescence is found in several different cardiovascular cell lineages and contributes to a range of age-associated cardiovascular disease pathophysiologies including age-related myocardial remodelling, atherosclerosis, maladaptive remodelling post-myocardial infarction, hypertension and vascular dysfunction [49]. In the heart, senescent cardiomyocytes accumulate with age and have been implicated in the reduced resilience to myocardial stress seen with advancing age [50, 51]. In other parts of the cardiovascular system, the accumulation of senescent endothelial and vascular smooth muscle cells is associated with vascular ageing and vasomotor dysfunction as well as contributing to atherogenesis [52].

Senescence can also be induced by cardiovascular stressors independently of ageing, and senescent fibroblasts and cardiomyocytes are found in the area of myocardium affected by myocardial infarction [53–55]. Elimination of senescent cells ameliorates cardiovascular disease progression, reduces remodelling and increases function [51, 53, 56], improves vascular tone [57] and diminishes atherosclerosis plaque burden, number and size [58]. Human studies confirm increased myocardial and vascular senescence in individuals suffering from heart failure, coronary heart disease and hypertension [59–61]. Moreover, higher expression of many markers identified as SASP proteins in pre-clinical models including Fractalkine, GDF-15, IL-6 and TGF-β are associated with cardiovascular disease, cardiovascular-related mortality and poorer outcomes post-myocardial infarction in observational studies [62–65].

### Other conditions

Senescence has been implicated in multiple other conditions. Radiation and cytotoxic chemotherapy for cancer can both trigger cellular senescence, and in animal models, senolytic therapies can prevent or reverse the adverse consequences of these treatments [65]. Once a cancer has developed, some cells may enter a senescent state, which although preventing further replication in the short term may lead to treatment resistance and later escape from the senescent phenotype, promoting tumour recurrence [66]. An exhaustive list of other organ systems where senescence has been implicated in ageing-associated pathology is beyond the scope of this review, but senescence has been associated with chronic kidney disease, chronic liver disease and a range of lung diseases [67–69].

## Can we prevent or reverse senescence?

Studies using transgenic mice have shown that it is possible to eliminate p16Ink4a-positive senescent cells, and that doing so can reverse a wide range of age-related physiological changes and disease across multiple organ systems, increasing mouse median lifespan. This landmark proof-of-concept work shows that preventing or reversing senescence may be a viable strategy to counteract ageing and age-related disease [50, 70]. Importantly, the pleiotropic effects of senescence across multiple organ systems make this a particularly attractive target to improve overall health and fitness across the life-course and into old age.

### Exercise

Exercise has multiple beneficial effects on many of the fundamental biological mechanisms underlying age-related conditions [71] and beneficial effects on cellular senescence may underpin some of the benefits that exercise has on health and function. Senotherapeutic effects of exercise have been demonstrated in animal and human studies. These include positive effects on DNA repair mechanisms and immunosenescence, upregulation of anti-inflammatory cytokines, reduction in markers of systemic inflammation (including SASP markers), upregulation of telomerase activity and downregulation of apoptotic modulators, including p16INK4a and p53 [71, 72]. However, less is known about the cellular and molecular interactions between exercise and diet in preventing senescence.

### Nutrition

A growing number of dietary compounds with potential senotherapeutic properties have shown promise in cellular and animal models [73, 74]. These include the flavonoids quercetin, fisetin, curcumin and piperlongumine [75–78]. Quercetin and fisetin have both been shown to clear human senescent cells *in vitro*, and fisetin treatment significantly reduced senescent cells and SASP markers (IL-6, IL-8 and MCP-1) in human adipose tissue and other tissues in mouse models of ageing and senescence [76]. Other dietary compounds have been investigated for their suppressant effects on SASP without the induction of cell apoptosis (akin to senostatic drugs discussed below). These include resveratrol, kaempferol, apigenin (all flavonoids present in either fruits, vegetables or red wine) and epigallocatechin gallate, a phytochemical found in green tea [79]. Clinical trials of fisetin are underway for several conditions including osteoarthritis, frailty and chronic kidney disease, and quercetin has been tested in combination with the tyrosine kinase inhibitor dasatinib (see below). An alternative therapeutic approach is to deliver plant-rich diets (which are high in flavonoids) such as the Mediterranean diet [80], which may have beneficial impacts on multiple mechanisms of ageing. These mechanisms may include (but are not confined to) interference with cellular senescence both via senolytic and senostatic effects [81], although whether such an approach can deliver long-term benefits on senescent cell accumulation and activity is open to question. More generally, the life-extending benefits of caloric restriction (which has been defined as reducing dietary energy intake below usual energy requirements whilst maintaining optimal nutrition [82]) seen in animal models may be due in part to reduced accumulation of senescent cells [83]; conversely, obesity appears to promote generation of senescent cells [84].

### Senolytic drugs

Senescent cells are resistant to apoptotic cell death and exhibit up-regulated pro-survival pathways, commonly known as senescent cell anti-apoptotic pathways (SCAPs). By targeting these SCAPs, it may be possible to specifically eliminate detrimental senescent cells from tissues, whilst keeping young proliferating cells unscathed [75], and it is these pathways that senolytic drugs seek to target. This work ultimately led to the discovery of the senolytic drug cocktail of dasatinib (D) and quercetin (Q) as noted above. Since its discovery, multiple pre-clinical studies have shown that this drug combination can prevent age-dependent functional decline in muscle, bone, brain, heart and liver in mice [46, 68, 85, 86].

Since the identification of D+Q, several additional drugs with senolytic properties have been uncovered that target different SCAP pathways [87]. Navitoclax and other inhibitors of the BCL-2 family have been shown to be senolytic, reducing senescent cell burden *in vivo* in mice and improving haematopoietic stem cell and cardiac function during ageing [4, 88]. To date, many other senolytic drugs have been identified though drug screens, including HSP90 inhibitors, p53 targeting compounds and cardiac glycosides [89, 90]. Emerging evidence suggests that the efficacy of senolytic drugs varies between different tissues, thus more than one agent may be required to deliver benefit across all organ systems.

There are now several clinical trials planned or in progress using senolytics, in particular repurposed drugs and nutraceuticals, such as D+Q and fisetin. The first uncontrolled clinical trial using senolytic drugs showed some improvement in physical function in idiopathic pulmonary fibrosis patients after treatment with D+Q [91]. Another clinical study showed that D+Q reduced senescent cell markers in patients with diabetic kidney disease, demonstrating for the first time that these drugs reduce senescent cell numbers in humans [92]. Larger randomised controlled trials are required to better understand the potential of senolytics as therapeutic interventions, to evaluate safety and off-target effects, and to identify at what points in the life-course these drugs have the most benefit with the lowest risk.

The immune system is able to clear senescent cells to some extent, although some senescent cells are able to evade immune surveillance and clearance [93]. In a parallel with cancer therapeutics, attempts are now underway to augment immunological mechanisms to clear senescent cells, both via vaccination approaches and by chimeric antigen receptor T-cell therapies. These approaches have been shown to be able to enhance clearance of senescent cells in mouse models [94], but human studies have not been undertaken to date.

### Senostatic/senomorphic drugs

Another strategy to therapeutically target senescence is to dampen the SASP without removing senescent cells. This class of therapies is commonly referred to as senostatics (sometimes called senomorphics). Ideally, these therapies should not interfere with pathways controlling the cell-cycle arrest component of cellular senescence as this holds at least a theoretical risk of promoting the development of cancer. Many senostatics identified to date act by interfering with regulators of the SASP, such as inhibitors of NF-κB, p38 MAPK, JAK/STAT and mTOR pathways [95–99].

There are several examples of existing drugs that have been shown to have beneficial effects in pre-clinical studies, which are senomorphic/senostatic. For instance, the mTORC1 inhibitor rapamycin, which is used clinically as an immunosuppressant, has been shown to at low doses to decrease the SASP [98, 100] and to extend healthspan and lifespan in mice [101]. Metformin, a widely used drug to treat type 2 diabetes, has been shown to suppress the SASP [102] and to improve healthspan and lifespan in mice [103]. Metformin use is also associated with reduced all-cause mortality and the occurrence of age-related diseases in patients with diabetes, suggesting that it may have beneficial effects across multiple disease states [104]—a hypothesis now under test in the TAME (Targeting Ageing by Metformin) clinical trial, a large randomised trial of healthy older people investigating whether metformin can prevent progression to a composite endpoint of age-related conditions [105].

### Potential challenges to senotherapeutics

Whilst senescent cells have been shown to be detrimental during ageing and age-related disease, they can also play important beneficial roles in tumour suppression, tissue repair, wound healing and development [106–110]. Therefore, targeting senescent cells may not be desirable in all circumstances. Moreover, since studies have shown that a senescent-like phenotype can also occur in post-mitotic cells, it is also unclear if eliminating non-replaceable post-mitotic senescent cells (e.g. neurons, muscle fibres and cardiomyocytes) or even eliminating replaceable senescent cells too quickly may adversely affect tissue structure, function and integrity [111]. For these reasons, senostatic therapies may be particularly helpful in ameliorating the adverse effects of senescent cells (and preventing contagion of senescence) without removing senescent cells where they may still fulfil important roles. Further work is also required to characterise different subsets of senescent cells more precisely; such approaches may enable us to distinguish populations serving useful roles from those causing harm.

## Testing anti-senescence interventions

Although anti-senescence interventions are being tested for single-organ conditions, the real power of this approach is its ability to tackle multiple age-related conditions across multiple organ systems. Assessing the pleiotropic effects of anti-senescence interventions therefore requires measurement of outcomes across multiple organ systems, preferably using measures that integrate multiple physiological functions [112]. Fortunately, this approach is one that aligns well with existing measurement approaches in geriatric medicine, where an emphasis on integrated measures of function (for instance frailty, walk speed or falls) and quality of life are familiar to both researchers and clinicians. Another important feature of senolytic therapies is that a relatively short course of senolytics can remove senescent cells, which then take time to reaccumulate: so-called ‘hit and run’ effects [113]. Short courses of therapy may therefore have profound longer-term effects and the trajectory of progression of organ dysfunction may continue to diverge many weeks or months after the cessation of therapy. Follow-up and outcome measures therefore need to be collected for considerably longer than the period of the anti-senescence intervention to fully evaluate these longer-term potential benefits. The potentially beneficial roles of senescent cells in wound healing and cancer suppression mean that the use of long-term anti-senescence therapies (e.g. in midlife and in young old age) will require careful follow-up over many years (perhaps decades) to ensure that these unwanted effects do not outweigh any benefits. The pleiotropic effects of anti-senescence interventions mean that side effects may be seen in any organ system, and appropriately broad collection and reporting of adverse events across all organ systems (regardless of the target condition under study) will be needed.

## Conclusion

Uncovering the biology of senescence is starting to yield insights into points of intervention to both remove senescent cells and to ameliorate their deleterious effects using exercise, nutrition and drug approaches. Despite the excitement surrounding senotherapeutics, they are not yet ready for clinical deployment. A great deal of work will be needed to test senotherapeutic interventions in human trials before it is clear whether these approaches are of net benefit for the prevention or treatment of multiple age-related conditions. The timing, dose and duration of senotherapeutic interventions all need further interrogation in human studies, and better ways of characterising both the presence of senescent cells in humans, and of evaluating the response to senotherapeutics are needed. The litmus test for this exciting new class of therapies will be whether they can improve quality of life, function and healthspan across the life-course rather than merely extending lifespan without quality. The outcomes that are measured in clinical studies need to reflect these aims if senotherapeutics are to gain the trust of clinicians, patients and the public.
